# Internal dosimetry for radioembolization therapy with Yttrium‐90 microspheres

**DOI:** 10.1002/acm2.12042

**Published:** 2017-01-24

**Authors:** Maryam Fallahpoor, Mehrshad Abbasi, Ali Asghar Parach, Faraz Kalantari

**Affiliations:** ^1^ Department of Nuclear Medicine Vali‐Asr Hospital Tehran University of Medical Sciences Tehran Iran; ^2^ Department of Medical Physics Shahid Sadoughi University of Medical Sciences Yazd Iran; ^3^ Department of Radiation Oncology UT Southwestern Medical Center Dallas TX 75235 USA

**Keywords:** dosimetry, dosimetry, microsphere, Yttrium‐90

## Abstract

The absorbed doses in the liver and adjacent viscera in Yttrium‐90 radioembolization therapy for metastatic liver lesions are not well‐documented. We sought for a clinically practical way to determine the dosimetry of this advent treatment. Six different female XCAT BMIs and seven different male XCAT BMIs were generated. Using Monte Carlo GATE code simulation, the total of 100MBq ^90^Y was deposited uniformly in the source organ, liver. Self‐irradiation and absorbed doses in lung, kidney and bone marrow were calculated. The mean energy of Yittrium‐90 (i.e., 0.937 MeV) was used. The S‐values and equivalent doses in target organs were estimated. The dose absorbed in the liver was between 84 and 53 Gy and below the target of 80 to 150 Gy. The absorbed dose in the bone marrow, lungs, and kidneys are very low and below 0.1 , 0.4, and 0.5 Gy respectively. Our study indicates that larger activities than the conventional dose of 3 GBq may be both required and safe. Further confirmations in clinical settings are needed.

## 1 Introduction

The treatment of inoperable liver metastases with microspheres and particles labeled with isotopes is a promising hope for a condition once was regarded untreatable.^1^, ^2^, ^3^, ^4^, ^5^ The procedure is evolving and the radiations to lungs, preserved liver parenchyma, and the bone marrow in repeated treatments are the limiting concern. Patient's dosimetry is possible with simulation methods before the initiation of the treatment or can be calculated after the treatment for follow‐up purposes. We previously used Monte Carlo code for certain conventional imaging and therapeutic agents including Technetium‐99m, Iodine‐131, and Samarium‐153 in different phantoms^6^, ^7^ and in patient. Recently ^90^Y became available to us and labeling of the microspheres and clinical trials are under development.^8^, ^9^, ^10^ There are different methods for internal dosimetry and imaging of the ^90^Y.^11^ In this study, we provide a radiation dose estimate to major target organs after treatment of liver metastases with the ^90^Y Microspheres. The results are important for us before proceeding to the clinical use of the therapeutic agent. We define the procedure so that internal dosimetry become practical for the future uses. We employed GATE (GEANT4 Application to Tomographic Emission)^12^ as a dedicated Mont Carlo code for nuclear medicine and XCAT^13^ as an up‐to‐date hybrid phantom.

## 2 Material and methods

**Figure 1:**
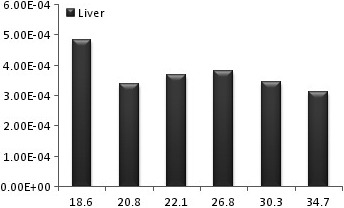
The diagram of liver equivalent dose for different female BMIs.

**Figure 2:**
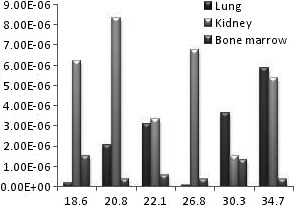
The diagram of equivalent dose of lung, kidney, and bone marrow for different female BMIs.

**Figure 3:**
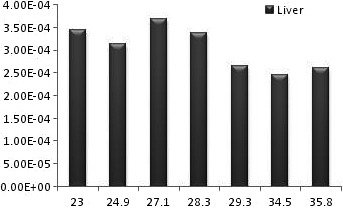
The diagram of liver equivalent dose for different male BMIs.

**Figure 4:**
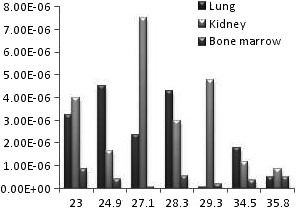
The diagram of equivalent dose of lung, kidney and bone marrow for different male BMIs.

Thirteen different total body XCAT phantoms including six female BMIs (18.6, 20.8, 22.1, 26.8, 30.3, and 34.7 kg/m^2^) and seven male BMIs (23.0, 24.9, 27.1, 28.3, 29.3, 34.5, and 35.8 kg/m^2^) were generated. The matrix dimensions were 128 × 128 × 600 with 0.3125 cm^3^ voxel sizes. We defined total activity of 100 MBq ^90^Y uniformly distributed in the liver as the source organ. Then, absorbed dose in four clinically important critical organs for the treatment by ^90^Y microspheres including liver, lung, kidney, and bone marrow were calculated. Simulations were performed with GATE, Monte Carlo code with the same method we previously documented.^6^ Among other physical interactions, attention has been given to various interactions of electron including Bremsstrahlung.^14^ The mean energy of ^90^Y, 0.937 MeV, was used for the beta particles. After dosimetry simulations with GATE, the output is two binary files containing the absorbed dose in voxels (in cGy) and the corresponding uncertainties respectively.^15^ We calculated the S‐values (in mGy/MBq‐s) for beta particles of ^90^Y according to Medical International Radiation Dose (MIRD) committee guideline.^16^ To report the results in a more familiar way, the corresponding amount of equivalent dose (in mSv/s) were shown as well (Fig. 1, 2, 3, 4).

## 3 Results

**Figure 5:**
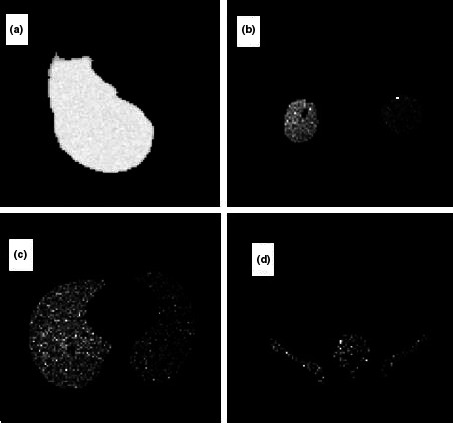
A view of organs selected as source and target organs; liver is both source of total of 100MBq Yttrium‐90 (uniform distribution) and the target organ (a); dose absorption in the lungs (b), kidney (c), and bone marrow (d) in other target organs are also visually depicted.

The successful uniform distribution of the tracer within the liver and the visual depiction of the deposited dose in the target organs are depicted in Fig. 5. Also the radiation to the main target organs is presented in Table 1 in males and females. In all studied BMIs the radiation to the liver is remarkably higher than the lung and other target organs by the power of at least 100 (Fig. 1 and Fig. 3). For an effective half‐life of the ^90^Y which equals to its physical half‐life^17^ (i.e., 64.1 hours) and theoretical fixed administered dose of 3 GBq, the total radiation doses to the liver in different BMIs of female phantoms range between 84 and 54 Gy while the radiation to the lung ranges between 0.34 and 0.005 Gy. The radiation to the liver in male phantoms ranges between 64 and 53 Gy and the radiation to the lung between 0.26 and 0.004 Gy. The radiation to the kidneys varies between 0.48 to 0.08 in females and 0.43 to 0.05 in males. The highest bone marrow absorbed dose is below 0.1 Gy for an effective half‐life of the ^90^Y. The equivalent dose within the liver is generally about 1 mSiv/h while the dose to the bone marrow is below 0.003 mSiv/h and the equivalent dose to the kidney and lung is below 0.03 mSiv in females (Fig. 2) and 0.027 in males (Fig. 4).

**Table 1:** S‐values (mGy/MBq‐s) calculated from Yttrium‐90 in different female BMIs

Source organ	Sex	BMIs	Target organs			
			Liver	Lung	Kidney	Bone marrow
Liver	Female	18.6	1.2085e‐004	1.6692e‐008	5.1755e‐007	1.2834e‐007
		20.8	8.5090e‐005	1.7210e‐007	6.9404e‐007	3.1386e‐008
		22.1	9.2387e‐005	2.5969e‐007	2.8099e‐007	4.6769e‐008
		26.8	9.5030e‐005	7.5511e‐009	5.6558e‐007	3.1865e‐008
		30.3	8.6134e‐005	3.0210e‐007	1.2805e‐007	1.1142e‐007
		34.7	7.8204e‐005	4.8910e‐007	4.4929e‐007	3.2973e‐008
	Male	23.0	8.6110e‐005	2.7157e‐007	3.3380e‐007	7.1842e‐008
		24.9	7.8633e‐005	3.7746e‐007	1.3852e‐007	3.4974e‐008
		27.1	9.2373e‐005	1.9722e‐007	6.2601e‐007	6.0759e‐009
		28.3	8.4301e‐005	3.5794e‐007	2.4988e‐007	4.6876e‐008
		29.3	6.6163e‐005	6.0479e‐009	3.9694e‐007	1.7263e‐008
		34.5	6.1598e‐005	1.5001e‐007	9.7855e‐008	3.1512e‐008
		35.8	6.5184e‐005	4.1659e‐008	7.1693e‐008	4.3151e‐008

## 4 Discussion

We documented the dose to the liver and some critical organs after ^90^Y‐Microsphere therapy for liver metastases. The ranges of the absorbed doses in the liver are generally less than the target therapeutic dose for tumoricidal purposes of at least 80 Gy.^1^ We put the therapeutic dose homogeneously within the liver,^18^ but for the real practice one or more hepatic artery divisions could be catheterized^19^ and hence the extent of the radiation would be probably higher.^1^, ^2^ Certain calculation methods are available to estimate the radiation to tumor and normal liver parenchyma.^20^ With the same method using the patient's SPECT/CT data the exact radiation to the hepatic metastases could be calculated. Such data could be acquired by the injection of macroaggregated albumin tagged with ^99m^Tc or ^111^indium before the administration of the therapeutic dose of ^90^Y‐Microspheres via the hepatic artery catheter.^21^ There are methods for internal dosimetry based on MIRD formalism. In these methods, the activity deposition is concrete and less‐flexible to match with the variations of the real activity in the patients.^18^ For example to assess the lung shunt, the activity should be distributed uniformly in the lungs in these methods. Employing the simulation methods the real patient's SPECT/CT data could be used for this purpose.^22^, ^23^

We also documented that although the dose to the bone marrow is generally low, the unwanted radiation into the bone marrow is unexpectedly high and about twice of others in thin patients with low BMIs; this would be the condition of many patients candidate for ^90^Y‐microsphere injection because they have suffered long time of the underling malignant pathology and they received many courses of chemotherapy and external beam radiations and have concomitant malnutrition. The doses need adjustment in these patients and the benefit of the treatment versus the possibility of bone marrow suppression should be re‐evaluated before proceeding to therapy.

In addition to the general radiation dependent side effects of ^90^Y‐microsphere including flu like syndrome and gastric ulceration, a more specific complication is radiation hepatitis. Post‐treatment liver absorbed dose may assist the clinician to make sophisticated decisions including creation of portosystemic shunts when hepatic failure is encountered after radioembolization. The other concern is radiation lung fibrosis due to lung shunting of the tracer. The extent of the lung shunting should be ascertained with injection of ^99m^Tc‐MAA. In this study the radiation to the lungs with no shunt was studied. The radiation to the kidney, lungs and bone marrow was presented as the sample critical organs but any organ of concern could be added into this list easily. We found that the radiation in the kidney and lugs are reasonably lower than the dose limitation of 15 to 30 Gy.^1^ The absorbed dose in the bone marrow was negligible and below the threshold for even minor side effects (> 2 Gy).^24^

There are few data available for comparison. The radiation to the tumor in real intra‐arterial hepatic radioembolization was calculated to be about 76 Gy to the tumor by equation based calculation and 120–180 by MIRD approach which is comparable with the findings of the current study.^25^, ^26^ The safe dose to non‐tumoral liver parenchyma is considered less than 40 Gy.^22^ The radiation to the kidneys and lungs secondary to treatment with ^90^Y‐Zevalin in patients with lymphoma unresponsive to chemotherapy are equal and much higher than the estimated dose in our study respectively. The distribution of the Zevalin and Intra‐arterial hepatic radioembolization are different but the radiation dose to the lung and kidneys are comparably acceptable for the radioembolization.^27^

The major shortcoming of the current study is the lack of simulation with real patient's data in different dose, metastasis location and employed spheres.^24^ In fact the current data provoke us to precede the current simulation method in real clinical cases to consider enhancement of the future therapeutic administered doses.

## 5 Conclusion

We provided the data of the radiation dose into the liver and certain critical organs after ^90^Y‐microsphere therapy. The dose to the liver with conventional administered dose of 3 GBq is lower than the therapeutic target and the unwanted radiation to the other viscera is very low. However, particular concern in the thin patients may be amended before ^90^Y‐microsphere therapy to avoid bone marrow suppression, escalation of the administered dose may be used safely. The results should be confirmed in clinical setting.

## Conflict of Interest

No conflict of interest exists in relation to this article.

## References

[1] Salem R , Thurston KG . Radioembolization with 90 Yttrium microspheres: a state‐of‐the‐art brachytherapy treatment for primary and secondary liver malignancies: part 1: technical and methodologic considerations. J Vasc Interv Radiol. 2006;17:1251–1278. 16923973 10.1097/01.RVI.0000233785.75257.9A

[2] Kennedy A , Coldwell D , Sangro B , Wasan H , Salem R . Radioembolization for the treatment of liver tumors: general principles. Am J Clin Oncol. 2012;35:91–99. 22363944 10.1097/coc.0b013e3181f47583

[3] van den Hoven A , Rosenbaum C , Elias S , et al. Insights into the dose‐response relationship of radioembolization with resin yttrium‐90 microspheres: a prospective cohort study in patients with colorectal cancer liver metastases. J Nucl Med. 2016;57:1014–1019. 26912436 10.2967/jnumed.115.166942

[4] Nijsen J , van het Schip AD , Hennink W , Rook D , Van Rijk P , Klerk J . Advances in nuclear oncology: microspheres for internal radionuclide therapy of liver tumours. Current medicinal chemistry. 2002;9:73–82. 11860349 10.2174/0929867023371454

[5] Lau W , Leung W , Ho S , et al. Treatment of inoperable hepatocellular carcinoma with intrahepatic arterial yttrium‐90 microspheres: a phase I and II study. Br J Cancer. 1994;70:994. 7947110 10.1038/bjc.1994.436 PMC2033550

[6] Fallahpoor M , Abbasi M , Kalantari F , Parach AA , Sen A . Practical nuclear medicine and utility of phantoms for internal dosimetry: XCAT compared with Zubal. Radiat Prot Dosimetry. 2016;doi: 10.1093/rpd/ncw115. 10.1093/rpd/ncw115 27247443

[7] Fallahpoor M , Abbasi M , Sen A , Parach A , Kalantari F . SU‐ET‐507: internal dosimetry in nuclear medicine using GATE and XCAT phantom: a simulation study. Med Phys. 2015;42:3451.

[8] Abedi M , Shirvani‐Arani S , Nabid MR , Bahrami‐Samani A . Synthesis and characterization of a novel acryl amide‐based yttrium imprinted sorbent via the ATRP approach for the preparation of medical‐grade 90Y. Radiochim Acta. 2015;104:117–129.

[9] Poorbaygi H , Aghamiri SMR , Sheibani S , Kamali‐asl A , Mohagheghpoor E . Production of glass microspheres comprising 90 Y and 177 Lu for treating of hepatic tumors with SPECT imaging capabilities. Appl Radiat Isot. 2011;69:1407–1414. 21723135 10.1016/j.apradiso.2011.05.026

[10] Nosrati Z , Khanchi AR , Sheybani S . Preparation of low‐density 90Y microspheres consisting of mesoporous silica core/yttria shell: a potential therapeutic agent for hepatic tumors. J Radioanal Nucl Chem. 2014;301:373–382.

[11] Walrand S , Flux GD , Konijnenberg MW , et al. Dosimetry of yttrium‐labelled radiopharmaceuticals for internal therapy: 86Y or 90Y imaging? Eur J Nucl Med Mol Imaging. 2011;38:57–68. 10.1007/s00259-011-1771-7 21484382

[12] Jan S , Santin G , Strul D , et al. GATE: a simulation toolkit for PET and SPECT. Phys Med Biol. 2004;49:4543. 15552416 10.1088/0031-9155/49/19/007 PMC3267383

[13] Segars W , Sturgeon G , Mendonca S , Grimes J , Tsui BM . 4D XCAT phantom for multimodality imaging research. Med Phys. 2010;37:4902–4915. 10.1118/1.3480985 PMC2941518 20964209

[14] Stabin MG , Eckerman KF , Ryman JC , Williams LE . Bremsstrahlung radiation dose in yttrium‐90 therapy applications. J Nucl Med. 1994;35:1377–1380. 8046497

[15] Parach A‐A , Rajabi H , Askari M‐A . Paired organs—should they be treated jointly or separately in internal dosimetry? Med Phys. 2011;38:5509–5521. 21992369 10.1118/1.3637493

[16] Bolch WE , Eckerman KF , Sgouros G , Thomas SR . MIRD pamphlet no. 21: a generalized schema for radiopharmaceutical dosimetry—standardization of nomenclature. J Nucl Med. 2009;50:477–484. 19258258 10.2967/jnumed.108.056036

[17] Kim Y‐C , Kim Y‐H , Uhm S‐H , et al. Radiation safety issues in Y‐90 microsphere selective hepatic radioembolization therapy: possible radiation exposure from the patients. Nucl Med Mol Imaging. 2010;44:252–260. 24899961 10.1007/s13139-010-0047-7 PMC4042917

[18] Dezarn WA , Cessna JT , DeWerd LA , et al. Recommendations of the American Association of Physicists in Medicine on dosimetry, imaging, and quality assurance procedures for 90Y microsphere brachytherapy in the treatment of hepatic malignancies. Med Phys. 2011;38:4824–4845. 21928655 10.1118/1.3608909

[19] Campbell AM , Bailey IH , Burton MA . Analysis of the distribution of intra‐arterial microspheres in human liver following hepatic yttrium‐90 microsphere therapy. Phys Med Biol. 2000;45:1023. 10795989 10.1088/0031-9155/45/4/316

[20] Ho S , Lau W , Leung T , et al. Partition model for estimating radiation doses from yttrium‐90 microspheres in treating hepatic tumours. Eur J Nucl Med. 1996;23:947–952. 8753684 10.1007/BF01084369

[21] Baranowska‐Kortylewicz J , Augustine SC , Wisecarver J , Tempero MA . Patient—Specific Dosimetry of Indium‐l ll—and Yttrium‐90‐Labeled Monoclonal Antibody CC49. J Nucl Med. 1997;38:512–516. 9098192

[22] Cremonesi M , Ferrari M , Bartolomei M , et al. Radioembolisation with 90Y‐microspheres: dosimetric and radiobiological investigation for multi‐cycle treatment. Eur J Nucl Med Mol Imaging. 2008;35:2088–2096. 18618108 10.1007/s00259-008-0857-3

[23] Lhommel R , Van Elmbt L , Goffette P , et al. Feasibility of 90Y TOF PET‐based dosimetry in liver metastasis therapy using SIR‐Spheres. Eur J Nucl Med Mol Imaging. 2010;37:1654–1662. 20422185 10.1007/s00259-010-1470-9

[24] Stewart FA , Akleyev AV , Hauer‐Jensen M , et al. ICRP publication 118: ICRP statement on tissue reactions and early and late effects of radiation in normal tissues and organs –threshold doses for tissue reactions in a radiation protection context. Annals of the ICRP. 2012;41:1–322. 10.1016/j.icrp.2012.02.001 22925378

[25] C‐yO Wong , Qing F , Savin M , et al. Reduction of metastatic load to liver after intraarterial hepatic yttrium‐90 radioembolization as evaluated by [18F] fluorodeoxyglucose positron emission tomographic imaging. J Vasc Interv Radiol. 2005;16:1101–1106. 16105922 10.1097/01.RVI.0000168104.32849.07

[26] Gulec SA , Mesoloras G , Stabin M . Dosimetric techniques in 90Y‐microsphere therapy of liver cancer: the MIRD equations for dose calculations. J Nucl Med. 2006;47:1209–1211. 16818957

[27] Wiseman GA , White CA , Stabin M , et al. Phase I/II 90Y‐Zevalin (yttrium‐90 ibritumomab tiuxetan, IDEC‐Y2B8) radioimmunotherapy dosimetry results in relapsed or refractory non‐Hodgkin's lymphoma. Eur J Nucl Med. 2000;27:766–777. 10952488 10.1007/s002590000276

